# MANUDB: database and application to retrieve and visualize mammalian NUMTs

**DOI:** 10.1093/database/baaf009

**Published:** 2025-02-21

**Authors:** Bálint Biró, Zoltán Gál, Zsófia Nagy, Juan Francisco Garcia, Tsend-Ayush Batbold, Orsolya Ivett Hoffmann

**Affiliations:** Group BM, Data Insights Team, _VOIS, Kerepesi str. 35, Budapest, 1087, Hungary; Agribiotechnology and Precision Breeding for Food Security National Laboratory, Department of Animal Biotechnology, Institute of Genetics and Biotechnology, Hungarian University of Agriculture and Life Sciences, Szent-Györgyi Albert str. 4, Gödöllő, 2100, Hungary; Agribiotechnology and Precision Breeding for Food Security National Laboratory, Department of Animal Biotechnology, Institute of Genetics and Biotechnology, Hungarian University of Agriculture and Life Sciences, Szent-Györgyi Albert str. 4, Gödöllő, 2100, Hungary; Agribiotechnology and Precision Breeding for Food Security National Laboratory, Department of Animal Biotechnology, Institute of Genetics and Biotechnology, Hungarian University of Agriculture and Life Sciences, Szent-Györgyi Albert str. 4, Gödöllő, 2100, Hungary; Department of Plant Biotechnology, Institute of Genetics and Biotechnology, Hungarian University of Agriculture and Life Sciences, Szent-Györgyi Albert str. 4, Gödöllő, 2100, Hungary; Department of Plant Biotechnology, Institute of Genetics and Biotechnology, Hungarian University of Agriculture and Life Sciences, Szent-Györgyi Albert str. 4, Gödöllő, 2100, Hungary; Agribiotechnology and Precision Breeding for Food Security National Laboratory, Department of Animal Biotechnology, Institute of Genetics and Biotechnology, Hungarian University of Agriculture and Life Sciences, Szent-Györgyi Albert str. 4, Gödöllő, 2100, Hungary

## Abstract

There is an ongoing genetic flow from the mitochondrial genome to the nuclear genome. The mitochondrial sequences that have integrated into the nuclear genome have been shown to be drivers of evolutionary processes and cancerous transformations. In addition to their fundamental biological importance, these sequences have significant consequences for genome assembly and phylogenetic and forensic analyses as well. Previously, our research group developed a computational pipeline that provides a uniform way of identifying these sequences in mammalian genomes. In this paper, we publish MANUDB—the MAmmalian NUclear mitochondrial sequences DataBase, which makes the results of our pipeline publicly accessible. With MANUDB one can retrieve and visualize mitochondrial genome fragments that have been integrated into the nuclear genome of mammalian species.

Database URL: manudb.streamlit.app

## Introduction

Mitochondrial DNA fragments are continuously integrated into the nuclear genome. This process is called NUMTogenesis [1], while the sequence fragments that are transferred to the nuclear genome are called NUMTs [2]. From an evolutionary point of view, NUMTogenesis is a form of endosymbiotic gene transfer [3]. The molecular and cellular mechanisms of NUMTogensis are not fully understood. However, there is an agreement among investigators that, damage of the mitochondrial membrane is one of the main prerequisites for NUMTogenesis [1]. This membrane degradation can be caused by factors like radiation [4], reactive oxygen species [5], and mutations [6]. The process of mitophagy is responsible for the disassembly of the damaged mitochondria [7, 8] and the recycling of mitochondrial components. In case of inadequate mitophagy, mitochondrial sequences can enter the cytoplasm. If these NUMT precursors move into the nucleus, they can be utilized by the non-homologous end joining repair mechanism at double-stranded breaks. This integration can cause cancerous transformation [9]. Besides their relevance in cancer biology, NUMTs have important applications in the field of phylogeny [10–12], forensic sciences [13], and other genomic analyses. For these reasons, NUMTs have been systematically described in several species [14].

We previously published a NUMT mining pipeline [14] to extract NUMTs from mammalian reference genomes. In the current study, we describe a database and associated functionalities to store and interact with the data that were generated by our computational pipeline.

## Data collection and organization

**Figure 1.**
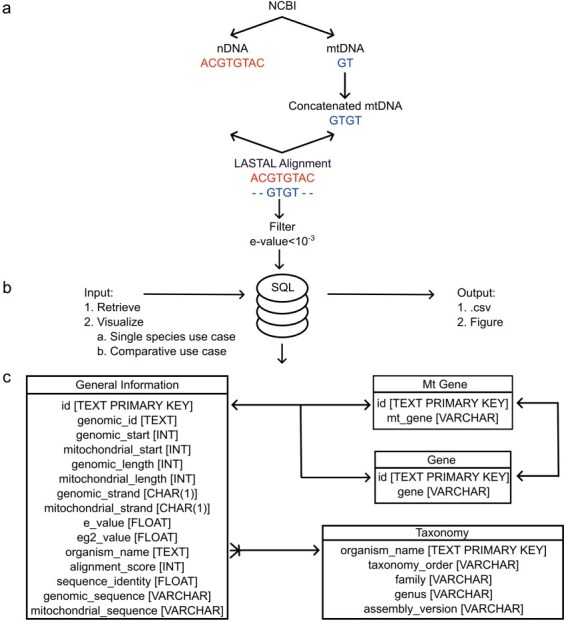
The architecture of MANUDB with the process of creating MANUDB’s data (a) (nDNA means nuclear DNA, while mtDNA means mitochondrial DNA); frontend and backend layers and detailed SQL schema with datatypes where arrows mean that the connected tables can be joined by the indicated fields.

**Figure 2.**
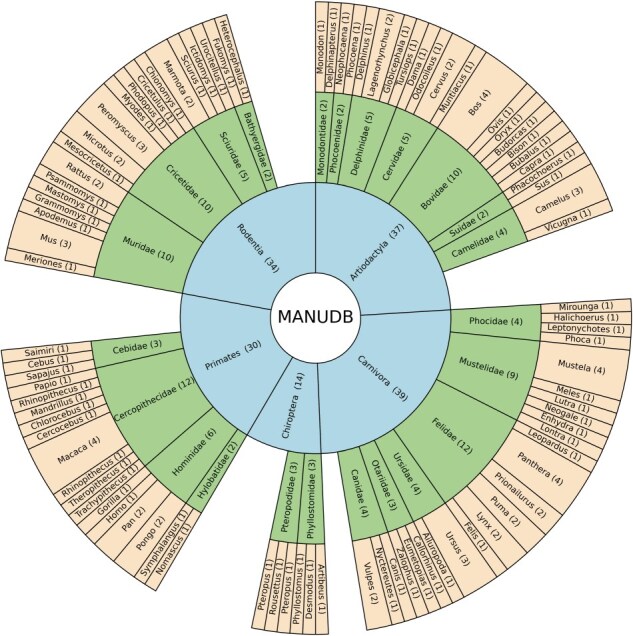
The taxonomical structure of MANUDB with order, family, and genus information where orders are represented by blue color, families are represented by green color and genera are represented by yellow color and numbers in the brackets display the sizes of the corresponding taxonomical units.

The data deposited in MANUDB (MAmmalian NUclear mitochondrial sequences DataBase) were constructed using sources that are associated with the National Center for Biotechnology Information—Reference sequence database (NCBI-RefSeq) [15]. Nuclear, mitochondrial genome sequences, and annotations were downloaded using the public file transfer protocol (FTP) sites of NCBI (https://ftp.ncbi.nlm.nih.gov/genomes/refseq/vertebrate_mammalian/ and https://ftp.ncbi.nlm.nih.gov/genomes/refseq/mitochondrion/). Taxonomy related data were also gathered from public NCBI FTP site (https://ftp.ncbi.nlm.nih.gov/genomes/GENOME_REPORTS/eukaryotes.txt). For the sequence alignments between the nuclear genomes and their corresponding mitochondrial genomes, LASTAL [16] was used with the parameters of +1 for matches, −1 for mismatches, 7 for gap opening, and 1 for gap extension. To overcome the boundary effect caused by the linearization of the mitochondrial genome, a concatenated mitochondrial sequence was used during the alignment. The alignment significance threshold was set to 10^–3^, which proved to be efficient in reducing false-positive rates. The procedure of using concatenated mitochondrial sequences with the above-mentioned LASTAL parameters and e-value settings has been published previously [17–19]. The data processing steps are visualized on Fig. 1a. After the alignment for each item e-value, genomic identifier, genomic start, mitochondrial start, genomic length, mitochondrial length, genomic strand, mitochondrial strand, genomic sequence, mitochondrial sequence, alignment score, and sequence identity information were extracted. Based on genomic coordinates, gene names (nuclear and mitochondrial), if any, were added as well. These data were further enhanced with order, family, and genus taxonomic information (Fig. 2). MANUDB currently contains 100 043 NUMTs which were extracted from 192 species that are categorized into 20 taxonomical orders, 65 families, and 116 genera. However, for the sake of interpretability, only the major orders and families are visualized in Fig. 2. We considered a taxonomical unit to be major if it has at least two taxa at one level lower in the taxonomical hierarchy.

## Database implementation and architecture

To facilitate future maintenance and provide a robust way of accessing the data, MANUDB is based on the SQLite serverless relational database management system [20] and its corresponding database engine (Fig. 1c). To reduce the storage requirements, SQLite was vacuumed. The user interface was programmed within the Streamlit ecosystem using additional HyperText Markup Language and Cascading Style Sheets to further improve usability. The backend and frontend are connected through Python scripts. The whole application is deployed within the Streamlit cloud. There are four Structured Query Language (SQL) tables in MANUDB, namely, General Information, Mt Gene, Gene, and Taxonomy (Fig. 1c). The General Information table contains information that correspond to a single NUMT. The Gene and Mt Gene tables contain the intragenic NUMTs and the NUMTs that cover annotated regions from the mitochondrial genome. The Taxonomy table contains information that correspond to a species. The General Information table can be joined with the Gene and Mt Gene tables in a one-to-one relationship, if applicable, while the General Information table can be joined with the Taxonomy table in a many-to-one relationship (Fig. 1c). Based on the user’s input, the query interacts with different tables of the SQL database (Fig. 1b). All the source codes have been deposited and are freely available on GitHub (https://github.com/balintbiro/MANUDB/).

## Functionalities

MANUDB currently supports two functionalities, namely, Retrieval and Visualization.

Using the Retrieval functionality, users are able to download specific datasets into .csv formatted files. Fields to retrieve can be selected based on the specific tables and fields of the SQL schema (Fig. 1c). Another option is to export the whole dataset with all the fields defined previously based on the specified species. The General Information table contains fields (e-value, eg2 value, sequence identity, and alignment score) that reflect the confidence of the actual alignment. These data offer the possibility to filter NUMTs based on their significance. Derived from the location of NUMTs, nuclear and mitochondrial annotations (gene names) have been added. These data are stored in the Gene and Mt Gene tables. Analyzing intragenic NUMTs can provide valuable insights about the genetic background of NUMT-associated diseases and the evolutionary patterns of NUMTogenesis. Additionally, information of the Gene and Mt Gene tables can help in mapping homologous NUMTs between species. We have made NUMT sequences (genomic and mitochondrial too) available in the General Information table. Integrating this sequence data with taxonomical information can shed light on homologous NUMTs. By exporting a part of MANUDB, users have the ability to conduct downstream analysis on the mammalian NUMTs of a species of interest.

By interacting with the Visualization functionality, users can generate and download publication-ready figures. We define two options for this functionality. One is the single species use case, while the other one is the comparative use case.

**Figure 3.**
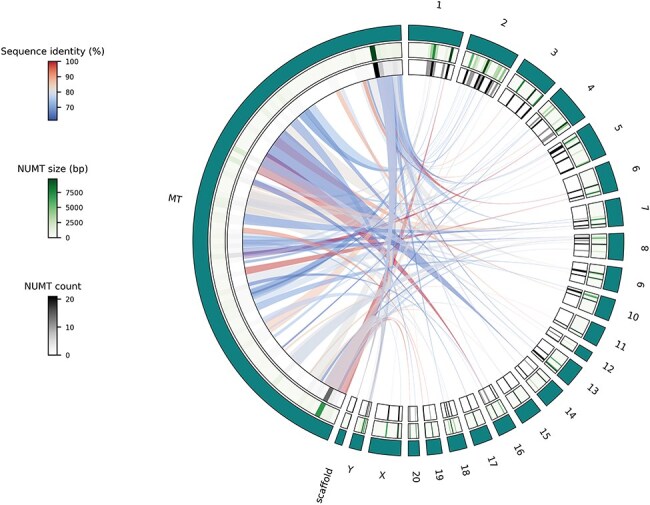
Circos plot of the Visualize functionality, single species use case using NUMTs of the rat genome where teal-colored outer track represents the different chromosomes and scaffolds; middle track shows a heatmap representation of cumulative NUMT sizes (in bp) of a given chromosome’s part while the inner track displays the number of NUMTs located in a specific part of a chromosome and links show the actual genetic flow from the mitochondrial genome to the parts of the nuclear genome.

The single species use case offers the possibility to display NUMTs of species of interest using Circos plots. The visualization itself is performed by using the Python implementation of Circos [21]. The output format of this functionality can be .png or .svg, based on the user’s preference. This type of visualization has proven to be intuitive and efficient [9, 22–24] to describe the genetic flow from the mitochondrion into the different parts of the nuclear genome. To demonstrate this Visualization functionality, the rat genome and its NUMTs were selected (Fig. 3). All the autosomes and sex chromosomes are plotted together with the mitochondrion. Unplaced and unlocalized scaffolds are merged and plotted as well. Scaffolds are merged together, since, e.g. in the rat genome, there are more than 150 unplaced and unlocalized scaffolds, which would be impractical to visualize individually. In some previously published studies, to reduce computational bias, scaffolds are merged or even omitted [25]. Heatmap representation of the cumulative size and numbers of NUMTs of different chromosomal regions are displayed using green and gray scales, respectively (Fig. 3). Links representing NUMTs are colored based on their sequence identity (relative to the whole NUMT) using a blue to red continuous scale from lowest to highest (Fig. 3).

**Figure 4.**
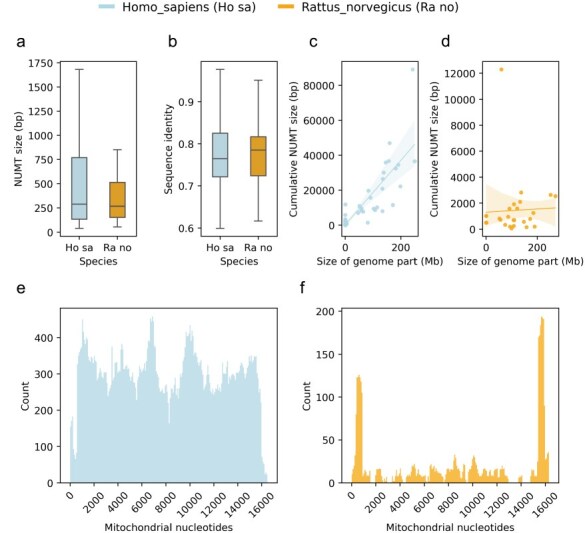
Plots of the comparative use case using the human and rat NUMTs as examples with distribution of NUMT sizes (a); distribution of sequence identities of NUMTs and their corresponding mitochondrial regions (b); regression plots of selected species’ genomic parts (chromosomes and scaffolds) and their corresponding cumulative NUMT sizes (c and d) and distributions of NUMTs in terms of mitochondrial nucleotides (e and f).

The comparative use case enables users to compare the NUMTs of two species using different visualizations (Fig. 4). This can be helpful if the users would like to place the NUMTs of their species of interest into the context of other genomes’ NUMTs. For this purpose, MANUDB displays the distributions of NUMT sizes of the selected species using boxplots (Fig. 4a). It also compares the sequence identities of the NUMTs and their corresponding mitochondrial regions with boxplots (Fig. 4b). In this case, the sequence identity is given as a ratio and so it varies between 0 and 1. The regression plots display the relationship between the size of a given genomic part (chromosome or scaffold) and the cumulative size of its corresponding NUMTs (Fig. 4c and d). The sizes of the genomic parts are displayed in bp. For the regression plots, MANUDB uses linear regression with 95% confidence intervals (shaded areas on plots). The confidence intervals are calculated using 1000 bootstrap iterations. The comparative use case of the visualization functionality provides plots for demonstrating the distribution of NUMTs along the linearized mitochondrial genome of the selected species (Fig. 4e and f). This type of visualization sheds light on the coverage of different mitochondrial parts.

## MANUDB’s testable hypotheses

In its current phase, MANUDB supports descriptive analysis of mammalian NUMTs with visualizations to make the complex genomic process of NUMTogenesis interpretable. We consider it a starting point for in-depth experiments as well as an interface for rapid fact checking. To demonstrate its potential usage as an exploratory platform, we examine the rat genome’s NUMTs.

We formulate three testable hypotheses. The first one is that the NUMTs of the rat genome originate from the whole mitochondrial genome. The second one is that the number of rat NUMTs is comparable with the number of human NUMTs as their nuclear genome sizes are similar. And our third testable hypothesis is that the NUMTs of the rat genome exhibit the same pattern as human NUMTs do, i.e. they tend to be longer in total on longer genomic elements (chromosomes or scaffolds).

To have an initial idea about the NUMTs of the rat genome, we can interact with the Visualize functionality’s single species use case. By looking at the resulting plot (Fig. 3), we can reject our first hypothesis. It is obvious that there are parts of the rat mitochondrial genome that do not contribute to NUMTs and so cannot be found in the nuclear genome. To investigate our second hypothesis, we can use the Retrieval or Export functionality. After exporting the NUMTs of our species of interest, we can see that there are 120 NUMTs in the rat genome, while there are more than 800 in the human genome. So, even though humans and rats have similar nuclear genome sizes, their NUMT contents are dramatically different. To test the third hypothesis, we can use the Visualize functionality’s comparative use case. After generating the comparative plots (especially C and D subplots), we can see that the size of a given genomic part and the cumulative size of its NUMTs do correlate in the human genome. However, it is not the case in the rat genome. Based on this, we can reject our third hypothesis too.

## Conclusions

In this paper, we have described MANUDB, which provides data originating from a computational pipeline in a structured and searchable way. This pipeline makes it possible to extract NUMTs from mammalian reference genomes. On top of this, two distinct functionalities were programmed as parts of MANUDB. With the Retrieval functionality, we can search MANUDB based on specified species. The result of the search is downloadable. The Visualize functionality helps us in understanding the genetic flow between the mitochondrial genome and different parts of the nuclear genome. The single species use case produces publication-ready Circos plots which can be downloaded. By interacting with the comparative option, users can place the NUMTs of their species of interest into the context of other species genomic environment which can help us to understand the evolutionary background of NUMTogenesis.

## Data Availability

The source code is available at https://github.com/balintbiro/MANUDB/ and MANUDB is available at manudb.streamlit.app.
